# Bioinspired Self-Shaping Clay Composites for Sustainable Development

**DOI:** 10.3390/biomimetics7010013

**Published:** 2022-01-10

**Authors:** Yuxiang Zhang, Hortense Le Ferrand

**Affiliations:** 1 Queen Mary Engineering School, Northwestern Polytechnical University, Xi’an 710072, China; yuxiang.zhang@se18.qmul.ac.uk; 2 School of Mechanical and Aerospace Engineering, Nanyang Technological University, Singapore 639798, Singapore

**Keywords:** bioinspiration, morphing, clay composites, sustainability

## Abstract

Bioinspired self-shaping is an approach used to transform flat materials into unusual three-dimensional (3D) shapes by tailoring the internal architecture of the flat material. Bioinspiration and bioinspired materials have a high potential for fostering sustainable development, yet are often fashioned out of expensive and synthetic materials. In this work, we use bioinspiration to endow clay with self-shaping properties upon drying. The composites created are based on clay and starch, and the internal architecture is built using celery fibers. The viscosity, shrinkage, and bending of the architected composite monolayers are studied for several compositions by measuring penetration depth and using optical characterization methods. Bilayer structures inspired from plants are then processed using a simple hand layup process to achieve bending, twisting, and combinations of those after drying. By layering a mixture of 32 vol% clay, 25.8 vol% starch, and 42.2 vol% water with 40 wt% embedded aligned celery fibers, it is possible to obtain the desired shape change. The work presented here aims at providing a simple method for teaching the concept of bioinspiration, and for creating new materials using only clay and plant-based ingredients. Rejuvenating clay with endowed self-shaping properties could further expand its use. Furthermore, the materials, methods, and principles presented here are affordable, simple, largely applicable, and could be used for sustainable development in the domain of education as well as materials and structures.

## 1. Introduction

Bioinspired self-shaping is an approach used to create structures with 3D shapes that are not easily obtainable using conventional manufacturing. The material is initially produced flat and an external trigger such as temperature or humidity is applied that deforms the flat piece into a 3D shape. To obtain a predictable and programmable final shape, the internal architecture and composition of the flat material has to be controlled to produce the desired deformation after applying the trigger. Thanks to bioinspired self-shaping, ceramic pieces with unusual twisting shapes can be encapsulated into vessels [1], hydrogels can be made to adopt shapes as complicated as natural orchid flowers [2], and flat wooden grids can rise into arcs [3]. Beyond creating 3D shapes out of flat objects, bioinspired self-shaping has also been leveraged to create materials that respond to external stimuli without the need for a direct electrical power input [4,5,6]. This approach has yielded sustainable, adaptive, and autonomous structures for robotics [7,8,9], medicine [10,11,12], and architecture [13,14], among other applications [15]. However, these morphing structures are generally fabricated using methods that demand specialized equipment, education, and resources such as 3D printing [16,17], and/or employ materials such as epoxy and other non-sustainable materials [18]. To use bioinspired self-shaping to create structures for sustainable development, local, sustainable materials should be used and processed via simple, local, and affordable means. One material that is cheap, commonly available and abundant world-wide is clay. Clay has been used for buildings, packaging, cooking, cooling, etc., for thousands of years. Today, clay is still one of the major materials used in construction in the form of bricks [19]. To rejuvenate clay-based materials and increase their range of application and attractiveness to the private and public sectors, clay could be endowed with smart properties such as self-shaping.

Simple and affordable solutions have been provided to enable facile and cost-effective fabrication of self-shaping structures, e.g., using hand crafting of paper, wood, plastic foil [20,21]. Also, biobased materials from protein, wood, and biobased polymers have been successfully employed for self-shaping [17,22,23]. Clay is a material that can be processed via various, simple methods. For example, clay mixtures can be prepared to allow extrusion and 3D printing without the need for extensive equipment [24,25]. Moreover, clay-based composite materials have shown increased mechanical properties as compared with raw clay, and can be used as moisture control in buildings [26,27,28,29]. Change in shape in clay-based materials is ubiquitous under the form of warping. Warping occurs naturally in thin clay plates during drying and leads to bending at the corners of the plates [30,31]. Warping is generally not desirable, since it is difficult to control and leads only to bending.

**Figure 1:**
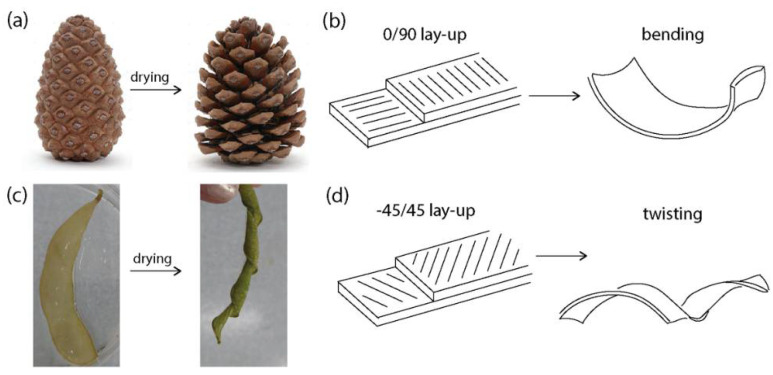
Principle of bioinspired self-shaping. (**a**) Pictures of a pinecone before and after morphing upon drying; (**b**) Schematics of the 0/90 layup architecture in the pinecone scales, leading to bending; (**c**) Pictures of an edamame seed pod before and after morphing upon drying; (**d**) Schematics of the −45/45 layup architecture in the seedpod, leading to twisting.

To enable programmable self-shaping of clay-based materials, a bioinspired approach could be used. Bioinspired self-shaping is inspired by the morphing of plants where the cellulose fibers embedded in the plant’s internal structure give anisotropic mechanical properties and deformation upon swelling and drying [32,33] (Figure 1). For example, the scales of pinecones can be schematized as a bilayer architecture with stiff cellulose fibers oriented at perpendicular directions between the two layers (Figure 1a,b). When the hydration level increases, the matrix swells predominantly in the direction perpendicular to the fiber orientation. Due to the bilayer architecture in a so-called 0/90 layup, where 0 and 90 are the angles of the fiber’s orientation in the bottom and top layers, the overall composite bends when hydrated. In other natural structures such as the edamame pod, the cellulose fibers form a −45/45 layup leading to twisting upon change in hydration (Figure 1c,d). The morphing of plants has been reproduced in synthetic materials by controlling their internal architecture with long fibers arranged into similar 0/90 or −45/45 layups [34,35]. Furthermore, recent works have been using short particulates in the form of microfibers and microplatelets to locally tune the architecture and achieve a larger design freedom and more shapes than long fibers can produce [2,36,37]. Bioinspired self-shaping in ceramic materials is scarce but has been demonstrated in technical alumina ceramics, and silica [1,38].

In this paper, we explore how to endow clay with self-shaping properties using bioinspiration and simple, affordable fabrication methods. First, the materials and strategy chosen are explained. Our study employs mixtures of clay with starch hydrogels and natural plant fibers to create anisotropic shrinkage. Then, we determine the composition of the matrix to have both homogeneity, facile processing, and shrinkage upon drying. Using the selected matrix, we added plant fibers and quantified the anisotropy with respect to the fiber content. Finally, we devise proof-of-concept self-shaping structures with unusual shapes. The work presented in this study aims at the facile fabrication of smart sustainable materials which could be exploited for sustainable development. To date, there has been no study of self-shaping clay, and most self-shaping procedures employ costly methods or chemicals. As an example of application, our bioinspired self-shaping clay-based composites could be used in schools to introduce the concepts of composites, smart materials, bioinspiration, and self-shaping. Upon drying (under 100 °C) without decomposition of organic fibers, the composites did not produce any unpleasant smell or toxic substances. Therefore, the strategies are safe to be used in schools and suitable for education. Education around bioinspiration and its potential for sustainable development are indeed key for fostering innovation [39,40].

## 2. Materials and Methods

### 2.1. Materials

Commercially available brown clay (Yellow River Delta, Dongying, China) containing mostly SiO_2_ (49.2 wt%) and Al_2_O_3_ (39.7 wt%), potato starch (Beidahuang, Dalian, China), purified water, and celery fibers (containing 52 wt% water) with a diameter of approximately 1.0 mm and length of 100 ± 20 mm from fresh mature celery containing cellulose, polysaccharides, structural proteins, and aromatic substances such as lignin, among others, were used in this study. 

### 2.2. Clay-Water Mixtures Preparation

Clay and water were mixed homogeneously by manual stirring. The clay was first added to the container, then water was added. A glass rod was used to mix the mixture until there was no more clay agglomerates.

### 2.3. Clay-Starch-Water Mixtures Preparation

Starch hydrogels with different starch concentration were first prepared by adding potato starch to water at the desired amount and the solution was heated at 100 °C until gelatinization occurred. Then, clay was added to the hydrogels at room temperature and the mixture was manually stirred until homogeneous. The weight fractions $f_{w}$ and the volume fractions $f_{v}$ for the constituent *A* in the mixture were calculated using:

(1) $$f_{w\left(A\right)}=\frac{M_{A}}{M_{A}+M_{B}+M_{c}}$$

(2) $$f_{v\left(A\right)}=\frac{V_{A}}{V_{A}+V_{B}+V_{c}}=\frac{\frac{M_{A}}{\rho_{A}}}{\frac{M_{A}}{\rho_{A}}+\frac{M_{B}}{\rho_{B}}+\frac{M_{C}}{\rho_{C}}}$$

where component *A*, *B*, and *C* can be clay, starch, or water. The densities for clay, starch, and water were estimated at 1.7 [41], 1.22 [42], and 1.0 g/mL, respectively.

### 2.4. Measurement of the Penetration Depth λ

**Figure 2:**
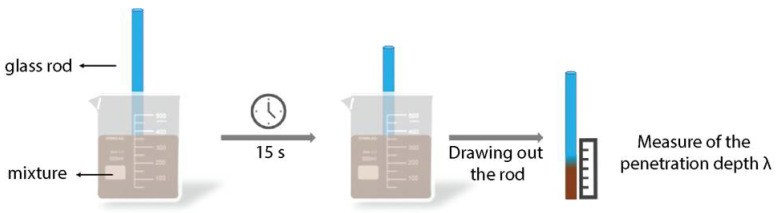
Schematics of the method for measuring the penetration depth λ.

The penetration depth λ was used as a measurement of the viscosity. Figure 2 is a schematic presentation of the testing process. In a penetration depth test, a weight was placed on the surface of the measured paste and made to penetrate the test sample by gravity. The depth achieved in a specified time, under defined conditions, is measured and used as an index, penetration depth λ. Due to the different rheological properties of different types of matrices, the weight used in testing varied; for instance, glass rods with a weight of 12 g each were used for pure clay compositions and the starch gel, whereas glass rods with a weight of 30 g each were used for the clay-starch mixtures. The rod was placed vertically at the center of the mixture and left standing on its own for about 15 s during which the rod sank under its own weight. The paste was viscous. As a result, when the glass rod was left on top of the mixture, it was able to remain standing vertically without external force. After a time, i.e., 15 s, the rod sank due to the gravity. After drawing out the rod, the depth of penetration λ, which is the length on the rod that had remaining liquid on its surface, was measured with a ruler (a similar method is typically used to measure the cake thickness during the slip-casting of ceramics [43]). Each measurement was repeated three times on fresh mixtures, then averaged.

### 2.5. Measurement of the Shrinkage of the Matrix

Clay-starch-water mixtures at various concentrations were shaped into rectangular samples by extrusion from a manually held syringe (60 mL, Xuehu, Guangan, China). The syringe was filled with the mixture and then the mixture was extruded into filaments laid parallel to form a rectangular shape. Typically, the samples had dimensions of 30×15×4 mm^3^. The samples were then heated at approximately 100 °C for 1 h in a convection oven (Joyoung, KX12-V500XK, Hangzhou, China) for drying. The shrinkage was measured along the width and length of the samples using the following equation:

(3) $$S\;\left(\%\right)=\frac{\left(l_{F}-l_{I}\right)\times 100\%}{l_{I}}$$

where S is the shrinkage, $l_{F}$ the initial dimension, and $l_{F}$ the dimension after drying. All measurements were repeated three times on separate samples.

### 2.6. Fabrication of Clay-Starch-Fiber Anisotropic Composites

To prepare clay-starch-fiber anisotropic composites, the clay-starch-water mixtures were used as a matrix and celery fibers were used as the anisotropic component. The long fibers were obtained by ripping celery stems. The fibers were cut at the desired dimensions of the composites. A mimic of hand layup lamination method was used for making the composite samples. First, a rolling pin was used to roll the clay-starch-water mixture into a thin film on a polyethylene substrate (0.1 mm, Miaozi, Zhoushan, China). Then, the film was cut into a rectangle and celery fibers were manually placed onto it. The roller was applied again to press the fibers and embed them in the matrix. A polyethylene plastic film was used to prevent the sample sticking on the rolling pin. The fiber content was varied from 20 to 40 wt%. Bilayer samples were processed in the same way, with sequential layering of the two layers. 

### 2.7. Characterization of the Change in Shape

The change in shape of the composites was triggered by drying them at 50 °C for 40 min, followed by 80 °C for 30 min. Pictures of the samples were taken with a standard camera and the curvatures of the samples were measured using a Fiji plugin for curvature analysis, Kappa in ImageJ. The curvatures were measured by tracing an initial shape with a B-Spline curve and then fitting that shape to the image of the cross-section of the sample with a minimization algorithm in the plugin.

## 3. Results

### 3.1. Approach

**Figure 3:**
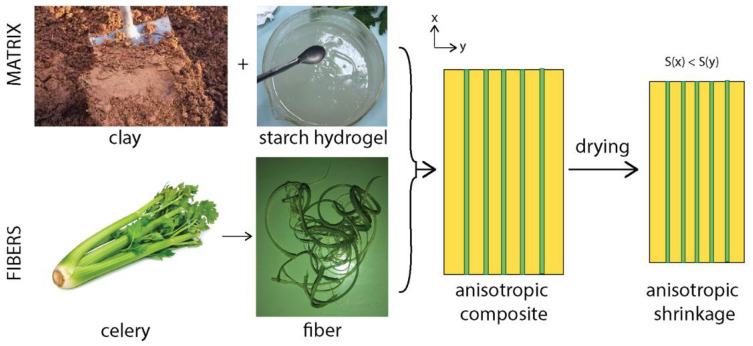
Fabrication approach for morphing clay-based composites. The matrix is composed of a mixture of clay and starch hydrogel to provide shrinkage after drying, whereas long fibers extracted from celery are employed to induce anisotropy. The clay-starch-fiber composite unit at the base of the morphing structure consists of the clay-starch matrix with embedded celery fibers aligned manually in a parallel direction to each other. The fibers are laid evenly on the matrix and the parallelism is measured by the naked eye. The distance between the fibers for each composite layer depends on the fiber fraction. For example, the distance for 25 wt% fiber composites in our study was approximately 0.5 mm, while the distance after drying shortened according to the shrinkage of the matrix. The anisotropy in the structure of the composite leads to anisotropic shrinkage S after drying. S(x) refers to the shrinkage along the x direction, S(y) along y.

The approach adopted to induce self-shaping and morphing using sustainable, commonly available materials, is to create an anisotropic composite using fiber reinforcement (Figure 3). This anisotropic composite is used as the base element in bilayers for programmable morphing. This approach is similar to that reported in other works on self-shaping materials and is inspired by the morphing of natural materials, such as wood, carnivorous plants, and seedpods [23].

A matrix composed of clay, starch, and water provides shrinkage upon drying. This is key because the volumetric change during drying is the trigger to the morphing. Celery fibers of a diameter of approximately 1.0 mm are used as the anisotropic reinforcements. Celery fibers are anisotropic and contain cellulose, polysaccharides, structural proteins, and aromatic substances such as lignin, among others [44]. Celery fibers have been used to reinforce biopolymers like poly lactic acid thanks to their high mechanical properties, with a tensile strength of up to 30 MPa [45,46]. They are also low cost, biodegradable, and easily available, making them ideal candidates for sustainable materials [45]. The anisotropic properties of the composites based on clay, starch, and celery fibers arise from the alignment of the celery fibers into an anisotropic architecture. Indeed, when the fibers are aligned along the direction x, they impede the shrinkage along this direction. As a result, the shrinkage *S*(*x*) along the direction x is lower than the shrinkage *S*(*y*), perpendicular to the fiber alignment direction.

The clay-based composites prepared in this work therefore employ solely natural and commonly available materials. Furthermore, a convenient method for fabrication of fiber reinforced composites is hand layup, which can also be applied for our compositions. In the next section, we determine the possible compositions to obtain a homogeneous and processable matrix based on clay, starch, and water.

### 3.2. Processing of Homogeneous Clay-Based Matrices

**Figure 4:**
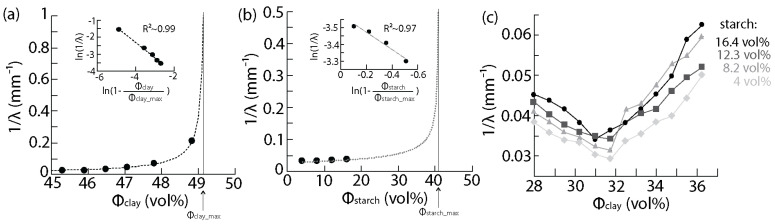
Effect of the matrix composition on the viscosity measured using the penetration depth method. (**a**) Inverse of the penetration depth 1λ as a function of the solid content in clay $\phi_{clay}$ in clay–water mixtures; (**b**) 1λ as a function of the solid content in starch $\phi_{stach}$ in the starch hydrogels; (**c**) Plot of 1λ as a function of $\phi_{clay}$ in clay–starch–water mixtures.

The preparation of homogeneous clay-based matrices requires proper mixing and adequate composition. The matrix should be liquid enough for its use in the hand lamination method but should not be so liquid that the shape cannot be retained or that extensive cracking occurs during drying. Indeed, being able to cut the matrix into the desired shape is necessary, and minimum cracking should occur during drying so as not to impede the potential future usage of the material. To determine the compositions that can be used for the matrix, the typical method is to measure its rheological properties. However, rheology requires a rheometer and knowledge of flow dynamics. In the context of sustainable materials processed in a sustainable fashion by the general public, simpler means are required. In our study, we therefore used the measure of the penetration depth λ from a solid glass rod placed onto the mixture, as a representative measure of the viscosity (Figure 4). The data recorded were fitted with a relation similar to the Krieger-Dougherty relation [47]. In short, the Krieger-Dougherty relation says that the viscosity η of a solid-liquid mixture increases with the concentration in solid φ until a maximum concentration $\varphi_{m}$ at which the viscosity diverges:

(4) $$\eta \left(\varphi \right)=\eta_{s}{\left(1-\frac{\varphi }{\varphi_{m}}\right)}^{-\left[\eta \right]\varphi_{m}},$$

where $\eta_{s}$ is the viscosity of the background liquid and [*η*] is a parameter that depends on the system. To verify that the inverse of the penetration depth can be used as a measure of the viscosity, we recorded the penetration depth λ for clay-water mixtures and starch-water hydrogels at increasing loadings in the clay and starch (Figure 4a,b). The points on the graphs are experimental data whereas the dotted line represents the fit using the theory. $\phi_{clay\_max}$ is the maximum attainable clay content. The inset is a plot of ln(1λ) as a function of $ln\left(1-\frac{\phi_{clay}}{\phi_{clay\_max}}\right)$ with experimental data and linear fit used to determine the parameters of the fit. Similarly, $\phi_{starch\_max}$ is the maximum attainable starch content. The inset is a plot of ln(1λ) as a function of $ln\left(1-\frac{\phi_{starch}}{\phi_{starch\_max}}\right)$ with experimental data and linear fit used to determine the parameters. The data could be fitted with the following formula, derived from Equation (4):

(5) $$\frac{1}{\lambda \left(\varphi \right)}=C∙{\left(1-\frac{\varphi }{\varphi_{m}}\right)}^{-\left[\eta \right]\varphi_{m}},$$

where C is a constant. The coefficients of correlation $R^{2}$ of the fits were of 0.99 and 0.97 for the clay and starch mixtures, respectively. $\varphi_{m}$ was determined experimentally as the concentration at which no more clay could be added. For the clay-water mixtures, we found that the maximum solid loading in clay that could be attained was $\phi_{clay\_max}$ = 49 vol%. For the pure starch hydrogels, the maximum solid loading was $\phi_{starch\_max}$ = 40 vol%. Moreover, a concentration in clay less than 45 vol% generally led to cracking after drying.

Mixtures containing clay, starch, and water at various loadings were thus prepared and their penetration depth measured for contents in the initial starch hydrogel, before introducing clay, varying from 4 to 16.4 vol%. (Figure 4c). The clay content is the content in the composite mixture.

It was found that for constant initial starch hydrogel concentration, the viscosity of the mixture first decreased with the increase in clay from 50 to 55 vol%, then increased for clay concentrations above 55 vol%. The decrease in viscosity with the increase in clay is thought to arise from the dilution of the hydrogel as the clay content is increased. Indeed, lower starch content leads to lower viscosity (Figure 4b). Then, above 55 vol% the effect of the dilution of the starch in the hydrogel is minimal and the viscosity rises with the clay content, as expected.

From these results, we have established that the measure of the penetration depth from a weight placed into the mixture is a suitable method for finding matrix compositions that are processable, provided that the shrinkage of the matrices is measured upon drying to select the right matrix composition for inducing self-shaping.

### 3.3. Shrinkage of the Clay-Based Matrices upon Drying

**Figure 5:**
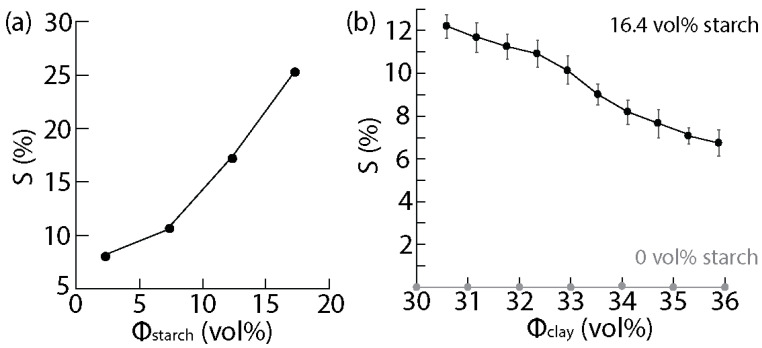
Shrinking of the matrix upon drying. (**a**) Shrinkage S as a function of the starch content in the pure starch hydrogel after drying; (**b**) Shrinkage S as a function of the solid loading in clay for the clay–starch–water mixtures with starch concentration of 16.4 vol% in the initial hydrogel suspension. In absence of starch, no shrinkage is recorded after drying.

To allow shape change upon drying, it is necessary to have a material that has its dimensions modified during drying. For this purpose, the shrinkage of the homogeneous mixtures after drying was measured for different matrix compositions (Figure 5).

The experimental results revealed that clay–water mixtures alone were not able to shrink sufficiently upon drying. Although clay is expected to undergo shrinkage, a large part of this shrinkage generally happens during the firing of the clay at high temperature, to transform the material into a ceramic. In the present case, it is possible that some shrinkage had occurred during the drying. Yet, no significant shrinkage could be measured, perhaps due to the small sample size or the lack of high-resolution instruments to measure accurately. To allow for more observable shrinkage, the mixtures containing the starch hydrogel were employed. Indeed, starch hydrogels contain a lot of water and shrink significantly during drying (Figure 5a). Unlike the clay, the hydrogels were elastic and did not crack when water was removed. The shrinkage increased with the content in starch. The starch hydrogel containing 16.4 vol% starch exhibited a homogeneous shrinkage of approximately 25%. Since this starch hydrogel was able to shrink significantly, it was selected for use in the clay–starch–water mixtures. After drying, the new clay-based matrices were able to shrink after drying, without the formation of cracks. The shrinkage was found to decrease as the clay content increased (Figure 5b). Similar to the viscosity, an increase in the clay content diluted the starch content, resulting in a lower concentration in starch. We already know that decreasing starch leads to decreasing shrinkage and that clay does not shrink, which explains our results.

Thanks to the addition of starch to the clay, a matrix that is able to shrink upon drying was obtained. However, the shrinkage was homogeneous whereas bioinspired shape-change requires anisotropic shrinkage. To induce anisotropy in the composites, we employed natural plant fibers and embedded them into the matrix using a method similar to lamination.

### 3.4. Building Anisotropy Using Fibers

**Figure 6:**
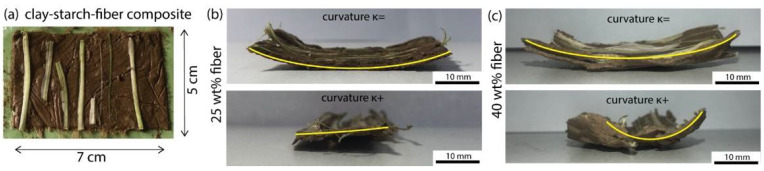
Anisotropic clay–starch–fiber composites. (**a**) Picture of the top view of a composite containing 32 vol% clay and 25.8 vol% starch laid up with parallel celery fibers of various lengths. Optical images of the composite monolayers after drying, highlighting their deformation for 25 wt% fiber content (**b**) and 40 wt% fiber content (**c**). In yellow are highlighted the curvatures of the monolayer samples with κ= the curvature along the fiber direction and κ+ the curvature perpendicular to the fiber direction.

Anisotropic clay–starch–fiber composites were obtained by manual hand layup using the clay–starch–water mixture containing 32 vol% clay and 25.8 vol% starch, and fibers extracted from celery (Figure 6). This composition was selected based on the penetration depth and shrinkage results. Indeed, 32 vol% clay had the lowest penetration depth, indicating high viscosity and formability. 25.8 vol% starch corresponds to the starch content in the composite mixture using the initial starch hydrogel of concentration 16.4 vol%, which showed high shrinkage of about 11%. The prepared composites typically had a rectangular shape and a thickness of approximately 2 mm (Figure 6a). The fibers were cut to have their length match that of the sample and they were positioned parallel to each other on the flat clay–starch film. Samples with various fiber content were prepared and dried and their shrinkage observed. Surprisingly, bending had already occurred in monolayer composite films (Figure 6b,c); in monolayers, bending deformation is not expected if the material is homogeneous along its thickness. However, in our process the fibers were embedded in the matrix by depositing them on top and using a rolling pin to push them down. As a result, the concentration of fibers is higher at the top surface of the composites than at their bottom. Since the celery fibers also contain water, it resulted in a higher shrinkage at the top of the composites than at the bottom. These mechanisms explain the development of a curvature in both directions, parallel, and perpendicular to the fibers, and why the curvature increased with the fiber content (Figure 6b,c). The influence of the substrate during the drying might further amplify the inhomogeneity in the thickness.

**Figure 7:**
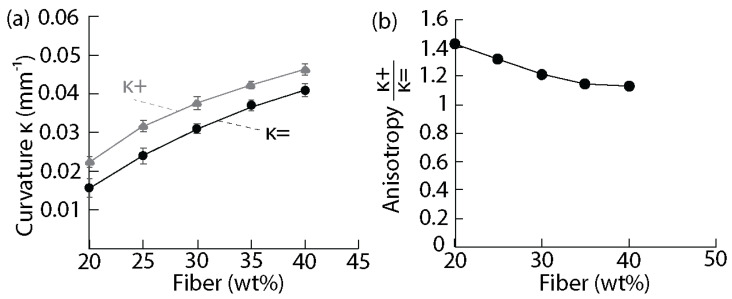
Curvatures and anisotropy in composite monolayers. (**a**) Curvatures κ= and κ+ as a function of the fiber content in the composites measured after drying; (**b**) Anisotropy in the deformation $\frac{\kappa +}{\kappa =}$ after drying as a function of the fiber content.

To explore the degree of deformation that can be obtained, anisotropic clay–starch fiber monolayer composites with various fiber contents were produced and their curvatures measured after drying (Figure 7). Contrary to the pure matrix shrinkage experiments, the drying of the composites was carried out in two steps: a first drying at 50 °C for 40 min followed by a second drying at 80 °C for 30 min. 100 °C drying could not be used for the composites because they led to the burning of the fibers. Instead, the low temperature of 50 °C was used to allow the fibers to lose water gradually. The final drying of the matrix was then performed at 80 °C.

The results show that a higher concentration in fiber increased the bending in the monolayer composites (Figure 7a). This is expected since individual celery fibers also contain water and had 52% weight loss after drying. A maximum fiber concentration of 40 wt% could be used in the composites. Above this concentration, there were too many fibers for the matrix, and they could not be held together anymore. Furthermore, we noted that the curvature perpendicular to the fibers was always higher than the curvature parallel to the fibers. This is expected due to the anisotropy in the architecture introduced by the fiber alignment. This anisotropy in curvature was found to increase when the content in fiber decreased (Figure 7b). This can be explained by the fact that more fibers increased the homogeneity of the material and reduced the shrinkage of the matrix. This phenomenon has been reported in other systems where the anisotropy in the shrinkage and deformation decreases when the filler content increases [48]. Moreover, it is likely that the reinforcing effect of the fibers also increases with the fiber content, reducing the deformability of the composite.

### 3.5. Bending and Twisting Deformations

**Figure 8:**
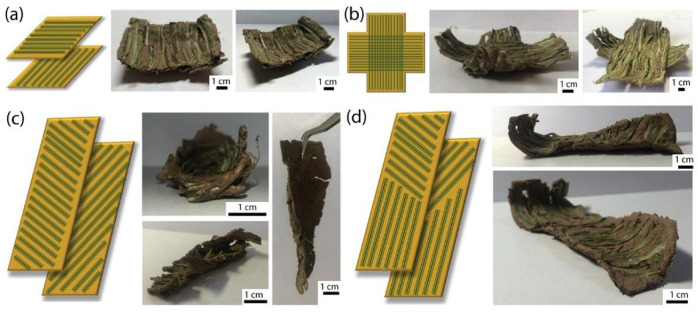
Clay-based samples with bioinspired self-shaping. (**a**) A bilayer sample with fiber aligned in perpendicular direction, bending in both directions; (**b**) A cross-shaped bilayer sample with fiber aligned in perpendicular direction, bending in the directions where fibers were aligned; (**c**) A bilayer with fibers aligned at 45° in two different directions, resulting in twisting; (**d**) A bilayer with fibers in the upper part were aligned at 45° cross and that in the lower part were aligned parallel.

To make use of the anisotropic shrinkage and to manifest the ability to create self-shaping sustainable clay-based materials, several proof-of-concept bioinspired specimens were prepared. The specimens changed their shape upon drying according to the design of their internal architecture (Figure 8). The composition used in these specimens was 32 vol% clay and 25.8 vol% starch with 40 wt% fiber content.

First, to mimic the bilayer structures that are commonly produced for bending, we prepared bilayers with perpendicular directions of fiber alignment (Figure 8a). After drying, bending was observed on all four sides, leading to a cup-like shape. Although this differs from other bilayer structures reported in the literature, this deformation is expected. Indeed, due to the fibers being located at the surface of each layer, deformation occurred at the four sides in single layers, as shown earlier. Here, the curvature increased and was symmetric at the four sides since the sample had a square, symmetric shape. Then, using a combination of bilayers and single layers, a cross-type of the object was produced (Figure 8b). After drying, the four branches of the cross bent upwards, as anticipated from its design.

Bending is interesting for creating unusual, complex shapes, but it can be obtained by simpler means. For example, a bilayer with a soft layer placed onto a stiffer layer would lead to bending upwards, despite each layer being homogeneous, without internal structuring. Furthermore, and especially with clays and ceramics, warping in thin films and layers often happens during the process, leading to the bending of the edges. This bending happens due to differential drying caused by a larger surface area at the edges [49,50]. To demonstrate why the use of architecture with fiber orientation is key in creating complex shapes, we created bilayer samples with fibers oriented at +/−45° (Figure 8c). After drying, extensive twisting was observed in the sample, as expected from this type of fiber orientation. The curvatures obtained were so strong that some cracking were visible in some parts of the sample. Cracking could potentially be reduced by slower drying, or by producing shapes with lesser deformations to reduce the internal stresses.

Finally, the last advantage of the fabrication method is the diversity in lay ups that can be produced. We created a bilayer composite with fibers oriented at +/−45° on one end and at 0/0° on the other end (Figure 8d). As predicted by this fiber arrangement, one side of the sample twisted after drying, whereas the other side bent.

## 4. Discussion

Our proof-of-concept examples showcase how bioinspired self-shaping in clay-based natural materials can be generated, using minimum technological tools and materials. Based on these principles and manual layup, a larger range of complex shapes could be fabricated to create sustainable alternatives for diverse usage. The final shapes could be later fired to yield ceramic objects and could also be scaled up for more applications. 

Up-to-date research on self-shaping ceramics usually involves costly approaches or chemical substances and there is no reported work on sustainable self-shaping clay. Ceramic objects with complex shapes are found for example in the textile industry with ceramic thread guides, in jewelry, arts, and architecture. The required curvatures of such pieces can be quite high. In our specimens, we achieved a variety of curvatures with bilayer samples typically in the range of 0.05 mm^−1^. Other work on bioinspired self-shaping alumina ceramics reported the variation of the curvature with the internal architecture of the specimen and with their geometry [1]. Thinner and longer shapes, for example, would exhibit higher curvature than thick and wide shapes. Furthermore, tuning the shrinkage would also control the curvature. In the alumina system, shrinkage was triggered by an ultra high temperature of 1600 °C and reached 25% in the direction perpendicular to the reinforcement. In our natural clay system, using high temperatures above 1000 °C could also lead to higher shrinkage since the firing of clay leads to densification and shrinkage depending on the clay composition and temperature [51]. 

Another approach to increase the curvature of our dried starch–fiber–clay composites could be to modify the composition. The method proposed in this paper could be easily extrapolated to other hydrogels and fibers. For example, common hydrogels used for self-shaping are thermoresponsive hydrogels such as poly N-isopropylacrylamide. This hydrogel could be rendered stronger by co-mixing it with polyacrylamide or alginate to reach a Young’s modulus of approximately 130 kPa [52,53]. By placing the hydrogels at 55 °C only, a curvature of about 0.12 mm^−1^ could already be achieved [54]. One particularity of thermoresponsive hydrogels is their shape-memory properties, which could be harnessed to induce reversible morphing in our clay composites. These hydrogels are chemical-based but are known to be biodegradable and largely used in medical applications. Reversible change in shape could be used for autonomous and adaptive sensing in response to changes in temperature and humidity. Such smart materials could improve the energy efficiency of buildings [55]. Other than using smart hydrogels, smart fibers could replace the celery fibers [56,57,58].

In the context of sustainable development, sustainable materials produced and used locally by laypeople can play a role in reducing the carbon impact of transportation and valorizing local resources. For example, clay tiles could be produced without a mold. This would reduce the need for gypsum mining, processing, and transportation, for example. Developing simple protocols as illustrated in this paper could lead to more innovation in the future (see Supplementary File S1). Sustainability, indeed, does not only include environmental impacts but has to take into account social and economic factors as well [59]. Empowering the population with simple, cheap, and applicable technical methods has been demonstrated to be key for entrepreneurship and sustainable development [60,61]. In architecture, it has led to new vernacular buildings culturally and environmentally adapted to their communities [62]. Contrary to the numerous starch–clay composites proposed in the scientific literature, where clay nanoparticles extracted from purified soil via complex processes are mixed with starch to form thin films [63], we employed natural clay which has been processed for thousands of years. Other natural or waste-based materials could be added to strengthen the composites, e.g., spent coffee grounds, fly ash, paper processing residues, cigarette butts, etc. [64,65]. In unfired clay, as reported in this work, durability in outdoor settings has been found to reach approximately eighteen months, highlighting the real potential impact of the material [66].

## 5. Conclusions

In this work, we have devised a composition for creating clay-based materials with bioinspired self-shaping upon drying. This was enabled by the shrinking of a starch hydrogel mixed with clay and the use of celery fibers that provided anisotropy. The largest bending curvatures parallel and perpendicular to the fiber alignment could reach 0.05 mm^−1^ and 0.04 mm^−1^, respectively. In addition, the samples exhibited tunable anisotropy values by varying the fiber content. The highest obtained anisotropy from bending was 1.4, underlining the importance of this anisotropy for the subsequent deformation. The fabrication method and the materials employed are deliberately simplistic to enable sustainable development potential at several levels. At the first level, natural, cheap materials can be used to teach the concepts of bioinspiration, composite processing, and shrinkage, among others. On another level, sustainable materials such as clay and plant fibers can be endowed with smart properties such as controllable self-shaping. On the final level, these materials can be sourced locally and used by local people, on-site, for domestic applications such as vernacular architecture. Not only is the cost of materials and fabrication minimal, but the process also brings back freedom of creation for laypeople and avoids issues such as large-scale manufacturing capabilities. Applying local materials for local applications has lately been widely investigated in the field of architecture. Therefore, the self-shaping strategy reported in this study could be applied to architectural materials, such as tiles, enabling a simple fabrication without requiring highly skilled workers or of specific molds. To push forward the use and application of this approach, the mechanical properties of the composites could be improved, for example, by adding other particulates to the clay. The composites could also be fired to retain their shape or the moisture level could be modified to create materials with adaptability.

## Data Availability

Data is contained within the article.

## Supplementary Materials

The following supporting information can be downloaded at: https://www.mdpi.com/article/10.3390/biomimetics7010013/s1, File S1: Protocol to produce bioinspired self-shaping objects from local clay, hydrogel, and plant materials.
