# Supplementary material for: Bioinspired Self-Shaping Clay Composites for Sustainable Development
Source: Biomimetics (Basel). 2022 Jan 10;7(1):13. doi: 10.3390/biomimetics7010013 (PMC8788514; doi:10.3390/biomimetics7010013)
Supplement: Supplementary file 1 [file biomimetics-07-00013-s001.zip › biomimetics-1541652-supplementary.pdf]

Supplementary Information to

Bioinspired Self-Shaping Clay Composites for Sustainable Development

Yuxiang Zhang [1] and Hortense Le Ferrand [2,*]

[1] Queen Mary Engineering School, Northwestern Polytechnical University, 710072, Xi'an, China; yuxiang.zhang@se18.qmul.ac.uk
[2] School of Mechanical and Aerospace Engineering, Nanyang Technological University, Singapore 639798, Singapore
* Correspondence: hortense@ntu.edu.sg

This file contains a protocol to produce bioinspired self-shaping objects from local clay, hydrogel, and plant materials.

1. Materials and Utensils

The following ingredients are required, in quantities relative to the intended dimensions of the products to fabricate:

- Clay
- Water
- Starch powder
- Fiber-rich plant such as celery

The utensils to be used are:

- A plastic foil, textile or similar. The foil will be used as a substrate to roll the material and should be easy to remove.
- A rolling pin and rollers. The thickness of the rollers should be the thickness of the desired layer, typically of less than 3 mm.
- A measuring beaker. A balance, measuring spoons, or similar, can also be used.
- A recipient for mixing the ingredients.
- A heater up to 100 °C.
- (optional) a drying oven with controllable temperature up to 100 °C.
